# The Malnutrition of Obesity: Micronutrient Deficiencies That Promote Diabetes

**DOI:** 10.5402/2012/103472

**Published:** 2012-03-15

**Authors:** Michael Via

**Affiliations:** Division of Endocrinology and Metabolism, Beth Israel Medical Center, Albert Einstein College of Medicine, 55 East 34th St, USA

## Abstract

Obesity and diabetes are increasing in prevalence worldwide. Despite excessive dietary consumption, obese individuals have high rates of micronutrient deficiencies. Deficiencies of specific vitamins and minerals that play important roles in glucose metabolism and insulin signaling pathways may contribute to the development of diabetes in the obese population. This paper reviews the current evidence supporting this hypothesis.

## 1. Introduction

Obesity is highly prevalent both in the United States and worldwide and is projected to surpass tobacco use as the most economically important modifiable risk factor in public health and disease [1–3]. Multiple genetic, environmental and behavioral factors contribute to the increasing trend in obesity [1, 3]. The increased availability of low-cost, high-calorie, nutrient-poor foods over the past four decades is a key component to the rise in obesity worldwide [3]. Modern agriculture and food processing techniques lead to a relative reduction in the micronutrient content of common foods [4]. Despite an excess of dietary calorie intake, obese individuals have relatively high rates of micronutrient deficiencies [5, 6].

The importance of certain micronutrients as cofactors in glucose metabolic pathways, pancreatic *β*-cell function and in the insulin signaling cascade suggests that deficiency in these micronutrients may play a role in the development of type 2 diabetes.

The risk of type 2 diabetes is increased 4-fold in obese patients [7]. The causal relationship of obesity and diabetes is complex. Increased insulin resistance, incretin hormone resistance, oxidative stress, pancreatic *β*-cell dysfunction, and genetic and behavioral factors all contribute to the development of diabetes in obese individuals [8]. Specific micronutrient deficiencies in obese individuals may also influence the development of type 2 diabetes.

## 2. Vitamin D

 High rates of vitamin D insufficiency and frank deficiency have been reported in obese individuals and in diabetics. The prevalence of vitamin D insufficiency (defined as <30 mg/dL) in obese individuals ranges from 80–90% [5, 9]. While some controversy exists over treatment targets in individuals with mild insufficiency of vitamin D especially for the purported extraskeletal effects of vitamin D supplementation, [10, 11] a significant amount of evidence suggests there may be some beneficial effect in using vitamin D supplementation for improvement in glucose metabolism and insulin signaling in patients with type 2 diabetes or impaired glucose tolerance [12].

 Preclinical evidence for the role of vitamin D in insulin secretion and function includes the presence of vitamin D receptors in human pancreatic *β*-cells, the detection of 1-*α*-hydroxylase activity, and insulin gene transcription responsiveness to vitamin D in pancreatic *β*-cells [13–16]. In vitamin D deficient animals, pancreatic *β*-cell dysfunction is noted, which is restored with vitamin D supplementation [17–19]. Many epidemiologic studies demonstrate an inverse relationship between 25-hydroxyvitamin D level and prevalence of type 2 diabetes [20–25]. Seasonal fluctuations in glycemic control in type 2 diabetics may also be due in part to fluctuations in vitamin D levels, though behavioral differences may also explain these findings [26, 27].

 Recent reviews and metaanalyses of clinical trials suggest a potential effect of vitamin D supplementation on the development of type 2 diabetes in high-risk individuals [12, 16, 28]. Three of five randomized trials demonstrate improvements in insulin sensitivity with vitamin D supplementation in subjects with insulin resistance or impaired fasting glucose [29–31]. One study showed improved oral glucose tolerance and insulin sensitivity in 71 obese males supplementation with 120,000 i.u. of cholecalciferol or placebo every 2 weeks after 6 weeks of followup [29]. A second trial showed improvement in insulin sensitivity and decreased fasting insulin levels in 81 vitamin D deficient subjects with baseline insulin resistance given 4,000 i.u. cholecalciferol daily or placebo [31]. In the third trial, subjects with impaired fasting glucose showed stability in fasting glucose levels after three years of daily supplementation of 700 i.u. of cholecalciferol compared to an increase in fasting glucose in the placebo group [30]. Two trials failed to show effect of vitamin D supplementation [32, 33]. One of these used only low doses of vitamin D (400 i.u. daily) that may not have been adequate; over 89% of participants did not achieve normal levels of vitamin D by the end of the trial [32]. Based on the results these five studies, patients at risk for development of type 2 diabetes may benefit from vitamin D supplementation.

The use of vitamin D in patients already diagnosed with type 2 diabetes has also been investigated. Of four recently published randomized trials, two demonstrate improvements in glycemic control [33–36]. In one trial, 92 early type 2 diabetics (defined by glycosylated hemoglobin ≥5.8% and 2-hour postprandial glucose ≥140 mg/dL) were randomized to receive 2,000 i.u. of cholecalciferol or placebo daily for 4 weeks [34]. Significant improvements in the measured glucose disposition index were noted in the treatment group. In another trial, 90 type 2 diabetic patients given 1,000 i.u. cholecalciferol or placebo daily for 12 weeks demonstrated decreased glycosylated hemoglobin by 0.4%, decreased fasting glucose by 13 mg/dL, improved insulin sensitivity, and 1-2 kg weight loss in the treatment group [35].

In one of the trials that demonstrated negligible effect of vitamin D supplementation in type 2 diabetics, patients given single dose of 300,000 i.u. of cholecalciferol found no change in fasting glucose, fructosamine, or insulin sensitivity despite normalization of plasma 25-hydroxyvitamin D levels [33]. However, adequate levels of serum 25-hydroxyvitamin D were not achieved in this trial. The average 25-hydroxyvitamin D level at the end of followup was 16 ng/dL.

The other negative study was a randomized trial with 6 weeks of followup in which 32 diabetic patients were given 40,000 i.u. of cholecalciferol or placebo every two weeks and showed no difference in glycosylated hemoglobin, fasting, glucose or insulin sensitivity [36]. Subjects in both treatment arms had a relatively high baseline 25-hydroxyvitamin D level in this trial (24 ng/dL), which may have contributed to the negative results.

 The use of vitamin D supplementation specifically for improvements in insulin sensitivity remains a controversial issue. There are presently ten ongoing clinical trials evaluating the use of vitamin D supplementation in type 2 diabetic patients and patients with impaired glucose tolerance, which may provide further guidance on this issue. Given the high rates of vitamin D insufficiency in the obese population and the observed benefits, clinicians may consider vitamin D supplementation in this population.

## 3. Chromium

 Chromium was recognized as an essential trace metal required in the insulin signal cascade through animal dietary experiments that were first performed in the 1950s [37, 38]. Molecular and cell culture studies demonstrate that chromium bound to the oligopeptide chromodulin enhances the tyrosine kinase activity of the insulin receptor and inhibits phosphotyrosine phosphatase activity, thereby amplifying the intracellular insulin signal cascade [39, 40].

Chromium deficiency in humans has been identified in severely malnourished patients who demonstrate severe insulin resistance, hyperglycemia, hypertriglyceridemia, and painful neuropathy. In published case reports, these symptoms and signs completely and rapidly resolve after the administration of chromium [41–44]. As a result of these findings, the inclusion of chromium in parenteral nutrition formulas has become standard [45].

The main dietary sources of chromium include yeast, meats, and wheat germ. The use of stainless steel pots and utensils increases the chromium content of food as traces of chromium are liberated from the steel during preparation. Body stores of chromium are intracellular, mainly in the liver. Individuals with type 2 diabetes have 20–40% lower blood chromium levels and 40–50% lower chromium levels measured in scalp hair compared to healthy controls [46–49]. Chromium deficiency rates in obese individuals are currently not available.

A number of clinical trials have investigated the role of oral chromium supplementation in patients with type 2 diabetes, insulin resistance, and the metabolic syndrome [50, 51]. In general, the results of these trials show only modest improvements in markers of insulin resistance and glucose metabolism. The greatest effects are noted in trials that administer higher doses and supplement with compounds such as chromium picolinate that provide a higher bioavailability of chromium. A review of 15 trials using chromium picolinate notes consistent improvements in glycemic control in 13 of the 15 trials, with an overall average decrease in glycosylated hemoglobin of 0.95% [52].

As with many supplements, the use of chromium supplementation in diabetic patients or in patients at risk for diabetes is controversial. The results of clinical trials that administer high doses of high bioavailability chromium suggest that supplementation with chromium may improve insulin resistance and glycemic control in diabetes [52]. Recently, the United States Food and Drug Administration release a Quality Health Claim regarding the use and safety of chromium picolinate for the treatment of insulin resistance [53]. This therapy may be beneficial in obese patients at risk for diabetes.

## 4. Biotin

 Biotin is a water soluble vitamin that serves as a cofactor for carboxylase enzymes in fatty acid synthetic pathways, the citric acid cycle, and aminoacid metabolism [54]. In addition to its biochemical function, circulating biotin regulates gene expression [55]. Prevalence data regarding rates of biotin deficiency in obese individuals or in diabetic patients are not presently available. However, patients with type 2 diabetes demonstrate lower circulating levels of biotin compared to healthy controls and an inverse relationship between biotin level and fasting plasma glucose has been reported [56, 57].

Animal studies demonstrate the development of insulin resistance in biotin deficient rats that resolves with biotin replacement [62]. At least part of the underlying mechanism of this phenomenon involves biotin-induced hexokinase gene expression that increases hepatic glucose uptake [63, 64]. Biotin also regulates transcription of the insulin receptor and improves pancreatic *β*-cell function [65, 66].

To date, only a few human trials evaluating the efficacy of biotin supplementation on glycemic control have been conducted. In one longitudinal study, diabetic patients treated for 28 days with 15 mg biotin supplements had improvements in fasting glucose and insulin levels [54, 67]. Two placebo-controlled trials studied the use of combined biotin and chromium supplementation in type 2 diabetic subjects [68, 69]. Improvements in glucose metabolism were noted in both trials. One trial demonstrated significant improvement in oral glucose tolerance and reduction in fructosamine after 4 weeks of followup [69]. The second trial demonstrated a reduction in glycosylated hemoglobin of 0.5% from a baseline of 8.7% in 447 subjects over 90 days [68].

 With the promising results of these early studies, the potential benefits of biotin supplementation may be considered in diabetic and obese patients.

## 5. Thiamine

 Thiamine is an essential micronutrient that acts as a cofactor for several key enzymes in glucose and aminoacid metabolism including transketolase, pyruvate dehydrogenase, *α*-ketoglutarate dehydrogenase, and *α*-keto acid decarboxylase [70]. The latter three are important regulatory enzymes that serve to mediate glycolysis, the citric acid and the pentose phosphate shunt pathways, respectively [70]. Thiamine deficiency leads to a relative reduction in these metabolic pathways and to increases in the polyol, hexosamine, protein kinase C, and advanced glycosylated end-product pathways of glucose metabolism that can lead to endothelial dysfunction and potentially worsen type 2 diabetes [71] (Table 1).

 Thiamine is absorbed in the proximal small bowel mainly through active transport [72]. The recommended daily intake of thiamine is 1.2 mg, approximately 0.5 mg per 1000 kcal consumed [73]. Important food sources of thiamine include pork, red meat, wheat germ, eggs, fish, and legumes [73]. Thiamine is virtually absent in food products containing refined carbohydrates such as milled rice and simple sugars, yet the metabolism of these foods requires relatively high amounts of thiamine and may lead to depletion [74]. In subjects on thiamine deficient diets, total body thiamine stores can be depleted within 2-3 weeks [74].

Severe deficiency of thiamine can lead to wet or dry beriberi or Wernicke encephalopathy, depending on the tissues involved. Moderate deficiency of thiamine may affect glucose metabolism and impact diabetes and related complications [71].

Thiamine deficiency measured by direct plasma levels of thiamine or by elevated erythrocyte transketolase activity has been observed in 15–29% of obese individuals planning to undergo weight loss surgery [75, 76]. The prevalence of thiamine deficiency has been reported in 17–79% of diabetic patients, although these studies include both type 1 and type 2 diabetic patients [77–79] (Table 1).

 Evidence for the effect of thiamine supplementation to reduce the risk and severity of type 2 diabetes has been demonstrated in a number of studies. Cultured endothelial cells demonstrate reduced production of reactive oxidative species and improved function in high glucose concentrations in the presence of thiamine [80, 81]. Both of these characteristics are expected to reduce the risk of diabetic complications and possibly reduce the risk of diabetes itself [8].

In a double-blind crossover trial, the administration of benfotiamine, a lipid soluble thiamine analogue, improved endothelial function, reduced markers of oxidative stress and reduced levels of advanced glycosylated end-products following a test meal in type 2 diabetic patients [82]. Amount of dietary thiamine intake has been correlated with levels of endothelial progenitor cells in type 2 diabetic patients [83].

 Long-term studies investigating the use of thiamine supplementation in diabetic patients have not been performed. The potential exists for thiamine supplementation to modify the course of diabetes by modulation of glucose metabolic pathways. Given high rates of thiamine deficiency in obese individuals and diabetic patients, supplementation may be considered. 

## 6. Antioxidant Vitamins 

 Despite importance of reactive oxygen species and increased oxidative stress in the development, progression, and associated complications of type 2 diabetes, trials using antioxidant treatment in both diabetic patients and patients with impaired fasting glucose have been largely disappointing and indicate little to no overall effect [84–86]. As a result, the routine supplementation with vitamin E, vitamin C, and vitamin A in diabetic patients is currently not recommended. 

 Vitamin E consists of a family of lipid soluble antioxidant compounds, of which *α*-tocopherol is the most abundant in the diet [87]. In contrast to other micronutrients discussed in this paper, obese and diabetic individuals have low rates of vitamin E deficiency. Studies in these groups fail to find any individuals that are vitamin E deficient [5, 9]. 

The use of vitamin E was studied in the Heart Outcome Prevention Evaluation (HOPE) trial, in which 3654 diabetic patients were randomized to receive 400 i.u. of *α*-tocopherol or placebo [88]. After 4.5 years of followup, no differences in cardiovascular outcomes, nephropathy, dialysis, or retinal laser therapy were seen between treatment groups. Several small trials show no differences in glycemic control with vitamin E supplementation in type 2 diabetics [89–92]. Although there are a few published trials that suggest modest improvements in glycosylated hemoglobin, [93, 94] a recent metaanalysis of 9 studies demonstrates no appreciable effect of vitamin E supplementation on glycemic control in type 2 diabetic patients [86]. 

 Vitamin C or ascorbic acid is a water soluble antioxidant vitamin that does not have significant body stores. Limited intake of fruits and vegetables can lead to rapid depletion [5]. Plasma vitamin C levels correlate inversely with body mass index and deficiency of vitamin C is reported in 35–45% of obese individuals planning to undergo bariatric surgery [58, 95]. Diabetic patients have lower dietary intake and lower plasma levels of vitamin C than healthy controls [96]. 

In a population study of 232,007 older adults, vitamin C supplementation was associated with mildly lower rates of type 2 diabetes in a dose responsive manner. Individuals that took 500 mg daily had a 9% reduction in prevalence of diabetes [97]. Data from the National Health and Nutrition Exam Study (NHANES) III cohort demonstrates significantly lower plasma levels of vitamin C in type 2 diabetics [98]. These results should be weighed carefully as population-based nutritional studies often have confounding factors. 

Few clinical trials have examined the use of vitamin C supplementation in type 2 diabetic patients. One small randomized trial in 20 diabetic subjects demonstrated that 1000 mg daily oral vitamin C supplementation improved glucose disposal rates after 4 months [99]. A second trial failed to show improvement in glucose metabolism or insulin resistance with 800 mg daily vitamin C supplementation [100]. In this study, plasma levels of vitamin C rose significantly; however, adequate plasma vitamin C levels were not achieved. 

Further studies in diabetic patients and in those at risk for developing diabetes may help to elucidate the role of vitamin C supplementation in this population. The high rate of vitamin C deficiency in obese individuals suggests that supplementation may be beneficial. Increasing dietary intake of fruits and vegetables can also address this deficiency and is currently recommended as part of a lifestyle intervention for the prevention and treatment of type 2 diabetes [101]. 

## 7. Conclusion 

As with nearly all biochemical processes, glucose metabolism and insulin signaling require cofactors and vitamins that are essential in the diet. Deficiencies in any of these micronutrients have potential to impair glucose metabolism and cause insulin resistance. Clinical evidence supporting this hypothesis regarding the metabolic effects of specific deficiencies including vitamin D, chromium, biotin, thiamine and vitamin C is mounting. Unlike vitamin E, which has little to no proven clinical effect when given as a supplement, these vitamins are known to be deficient at relatively high rates in obese individuals and in diabetic patients. Clinicians should consider addressing possible deficiencies of these micronutrients when advising obese patients who are at risk for the development of type 2 diabetes. The medical care plan for obesity should include lifestyle changes, healthy food choices with high-nutrient content foods as part of a balanced approach for the prevention of the development of type 2 diabetes. Use of specific vitamin supplements may adopted into this rational practice. 

## Figures and Tables

**Table 1:** Prevalence of micronutrient deficiencies in obesity and diabetes [5, 6, 46, 58–61].

Micronutrient	Prevalence of deficiency	
	Obesity	Type 2 diabetes
Thiamine B1	15–29%	17–79%^a^
Pyridoxine B6	0–11%	—
Cobalamin B12	3–8%	22%
Folic Acid	3-4%	—
Ascorbic acid C	35–45%	—^b^
Vitamin A	17%	—
Vitamin D	80–90%^c^	85–90%^c^
Vitamin E	0%	0%
Zinc	14–30%	—
Chromium	—	20–40%
Selenium	58%	—

—Prevalence data not available

^a^Data includes type 1 diabetic patients

^b^Decreased levels of ascorbic acid have been reported in diabetes

^c^Prevalence reflects rates vitamin D insufficiency.
